# Lumbar Apophyseal Ring Fracture With Central Disc Herniation: A Case Report

**DOI:** 10.7759/cureus.90771

**Published:** 2025-08-22

**Authors:** Eric Chun-Pu Chu, Benjamin Kah Chun Cheong, Eden YT Chu

**Affiliations:** 1 Chiropractic and Physiotherapy Centre, New York Medical Group, Hong Kong, CHN

**Keywords:** apophyseal ring fracture, chiropractic, disc herniation, fracture of the vertebral limbus, lumbar apophyseal ring fracture, lumbar posterior marginal node, posterior ring apophyseal fracture, posterior ring apophysis separation, posterior schmorl's node

## Abstract

Lumbar apophyseal ring fracture (LARF) with central disc herniation is a rare but important cause of neurological symptoms in adolescent athletes, often presenting diagnostic and therapeutic challenges. We report the case of a 14-year-old male professional tennis player who developed persistent bilateral lower extremity tightness and radiating sensations after intensive training, unresponsive to initial physiotherapy. MRI revealed an S1 apophyseal ring fracture with central disc herniation. With surgery declined, the patient underwent a multimodal chiropractic protocol, including targeted spinal manipulative therapy, shockwave treatment, and mechanical lumbar distraction, which contrasts with standard conservative management such as physiotherapy alone. This individualized approach resulted in complete symptom resolution and full return to competitive tennis. At 24-month follow-up, he maintained high function and satisfaction. This case highlights the importance of considering LARF in young athletes with refractory symptoms and demonstrates that, in selected cases, individualized nonsurgical management can achieve excellent long-term outcomes, in alignment with current evidence-based guidelines.

## Introduction

Lumbar apophyseal ring fracture (LARF) is a rare spinal injury, particularly in adolescents, and is often associated with lumbar disc herniation (LDH) [1-4]. The apophyseal ring, also known as the ring apophysis, is a secondary ossification center that forms along the periphery of the vertebral endplate, serving as the attachment site for the intervertebral disc via Sharpey’s fibers [3]. It is susceptible to fractures in adolescents due to its incomplete fusion and high biomechanical stress during activities like sports, which anchor the disc to the vertebra [3]. Among pediatric LDH cases, the prevalence of LARF ranges from 5.8% to 42%, with higher rates seen in young male athletes [4,5]. The most commonly affected sites are the L4, L5, and S1 vertebral endplates, reflecting the biomechanical stresses experienced during athletic activity [4].

LARF is increasingly recognized as a cause of neurological symptoms and low back pain in adolescents and may contribute to spinal deformity if not promptly addressed [4]. Diagnosis of LARF is challenging due to its rarity and nonspecific presentation, often requiring advanced imaging such as CT or MRI for accurate assessment [2]. While initial imaging is critical for establishing the diagnosis, the role of follow-up imaging remains debated. In some cases, repeat imaging can help monitor fracture healing or disc herniation resolution, potentially influencing the decision to continue conservative care or consider surgical intervention if findings worsen or fail to improve. However, in the absence of progressive symptoms or neurological deficits, clinical improvement alone may justify ongoing nonoperative management without routine follow-up imaging, thereby minimizing unnecessary radiation exposure and healthcare costs.

The literature on LARF concomitant with LDH in youth is limited, with ongoing debates regarding optimal management, ranging from conservative therapy to surgical interventions such as decompression, fragment removal, and spinal fusion, due to a lack of high-quality comparative studies [4]. While surgery is indicated in cases of persistent neurological deficit or failed conservative management [6], recent guidelines, including the 2023 WHO guideline for nonsurgical management of chronic primary low back pain, emphasize the importance and effectiveness of evidence-based, individualized conservative approaches in select patients, prioritizing multimodal strategies like exercise and manual therapies for adolescents without red flags [7]. This case report addresses these gaps by illustrating successful nonsurgical management in an adolescent athlete, contributing to the evolving consensus on tailored care for this rare condition.

## Case presentation

**Figure 1:**
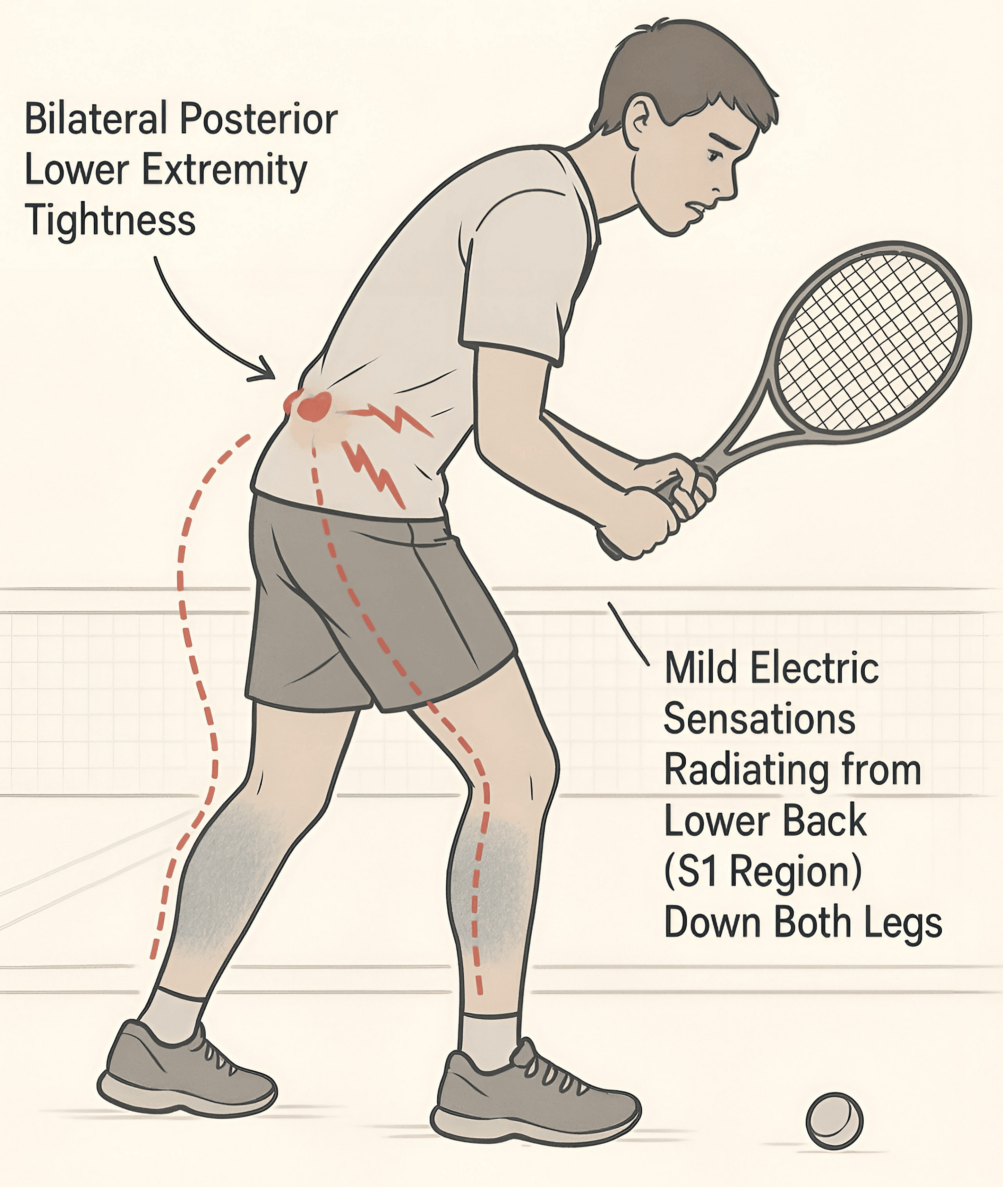
Schematic illustration of the presenting symptoms in a 14-year-old male tennis player with LARF at S1 and central disc herniation The posterior view shows bilateral posterior LE tightness (shaded regions), exacerbated by prolonged training or deep palpation. He had mild electric sensations radiating from the LB (S1 region) toward both legs (dashed red lines). LARF, lumbar apophyseal ring fracture; LB, lower back; LE, lower extremity Image credit: Eric Chun-Pu Chu, with the assistance of ChatGPT (OpenAI, Inc., San Francisco, USA)

**Figure 2:**
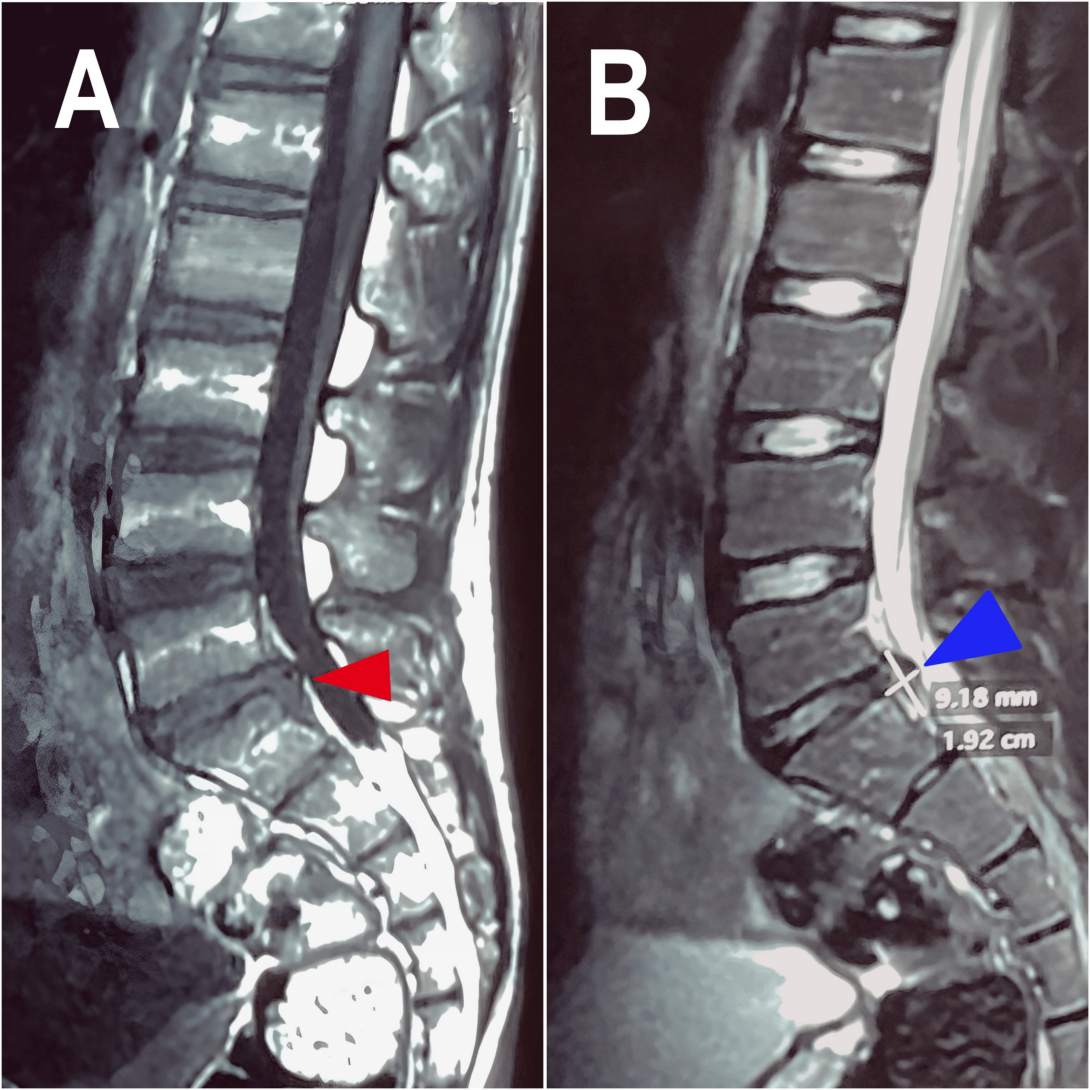
Sagittal T2-weighted MRI views of the lumbar spine (A) Suspected posterior ring apophyseal fracture at the S1 posterior vertebral body (red arrow), characterized by separation of a bone fragment at the posterior rim of the endplate, consistent with LARF. (B) Medium-sized central and paracentral disc extrusion at L5/S1 (blue arrow), measuring 1.9 cm in length, 0.9 cm in depth, and 2.5 cm in width. The extrusion results in moderate bilateral lateral recess narrowing with encroachment of both descending S1 nerve roots, as well as moderate bilateral exit foraminal narrowing without compression of the exiting L5 nerve roots. LARF, lumbar apophyseal ring fracture

A 14-year-old male professional tennis player presented with a six-month history of bilateral posterior lower extremity (LE) tightness accompanied by mild electric sensations radiating toward both legs (Figure 1). The patient reported that the symptoms began during his intensive tennis training routine and were initially thought to be related to overexertion. His sports team’s physiotherapist had diagnosed the issue as training-related muscle strain. The patient rated the stiffness and pain severity as 5/10 and noted that the symptoms were exacerbated by prolonged training sessions or deep palpation of the posterior LE. While no significant pain was reported in the lower back or LE, the symptoms were sufficiently disruptive to prevent him from competing in tennis matches. The patient denied numbness or weakness. The patient was evaluated by the sports physician and orthopedic surgeon, and an MRI revealed a LARF at S1 with a central disc herniation (Figure 2). Despite six months of physiotherapy with joint mobilizations, interferential current, soft tissue therapy, and core muscle training, there was no improvement in symptoms. Surgical intervention was recommended but declined by the patient’s parents, who opted for a conservative chiropractic approach.

Upon physical examination at the chiropractic clinic, the straight leg raise test revealed an inability to fully flex the knee due to tightness, with reproduction of symptoms at 60° bilaterally. Kemp’s test was negative. Hypertonicity and tenderness were noted bilaterally in the lumbar spine erector spinae, gluteal muscles, and quadratus lumborum, with palpation eliciting a pain response rated 4/10. Active range of motion of the lumbar spine showed restricted flexion at 30° (normal adolescent range: 60-80°), extension at 15° (normal: 25-35°), lateral bending at 20° bilaterally (normal: 30-40°), and rotation at 25° bilaterally (normal: 40-50°), with end-range tightness but no sharp pain. Functional assessments included a squat test limited to 50% depth due to posterior LE tightness and a single-leg balance test showing instability after 10 seconds on each side. Dermatome and myotome evaluations were normal, except for restricted knee flexion caused by tightness rather than weakness, with manual muscle testing graded 4+/5 for bilateral knee flexors. Segmental joint dysfunction was identified at C4-5, T4-6, L2-L5, and S1, with associated hypomobility on motion palpation. Baseline quality of life was assessed using the WHO guidelines.

At initial presentation, the differential diagnosis included common causes of back pain in adolescents, such as muscular strain, lumbar radiculopathy secondary to disc herniation, and growth plate injuries (e.g., apophysitis or spondylolysis). Muscular strain was considered unlikely due to the presence of neurological symptoms and lack of improvement with rest. While lumbar radiculopathy can result from an isolated disc herniation, imaging revealed the presence of an apophyseal ring fracture, distinguishing this case from typical disc pathology. Growth plate injuries and spondylolysis were also considered, but imaging findings were not consistent with these entities, supporting the diagnosis of LARF with associated disc herniation.

**Figure 3:**
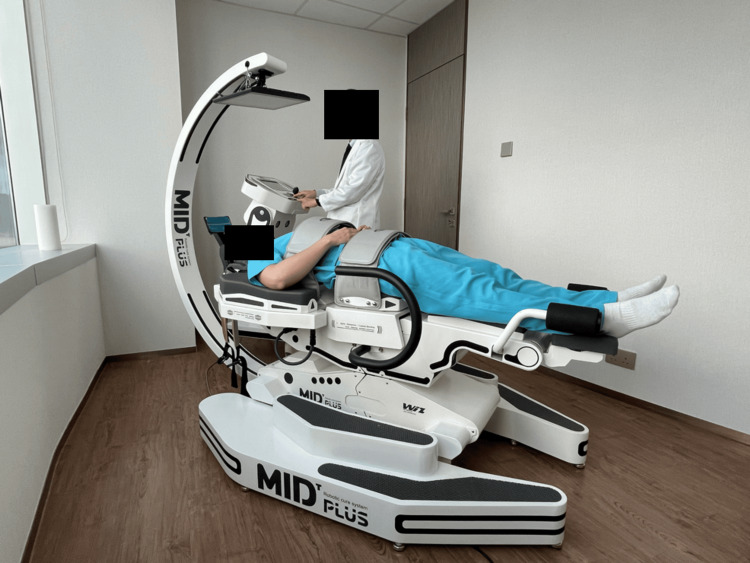
Patient positioning for mechanical lumbar traction targeting the L5/S1 disc space The patient is positioned supine on the spinal decompression table (28 kg force, L5-S1, 40:30 hold-to-rest ratio, 5° aslope) for 15 minutes per session, with a bolster supporting the lumbar spine to maintain natural lordosis and enhance comfort. Adjustable thoracic and pelvic harnesses stabilize the upper body and lower torso, facilitating controlled axial distraction to alleviate intradiscal pressure at the L5/S1 level.

Given the confirmed fracture and herniation, high-force manual therapy techniques targeting the lumbar spine or sacrum were avoided. Instead, a conservative treatment plan was initiated, starting with spinal manipulative therapy at the cervical and thoracic regions; shockwave therapy to the bilateral posterior knees; and mechanical lumbar distraction (28 kg force, L5-S1, 40:30 hold-to-rest ratio, 5° aslope) for 15 minutes per session (Figure 3). During the initial phase, the patient underwent treatment three times a week for four weeks, resulting in a 50% reduction in symptoms. The frequency of treatment was then reduced to two times per week, with additional core muscle strengthening exercises incorporated into the care plan.

After six months of consistent treatment, the patient experienced complete resolution of his symptoms and was able to return to competitive tennis without limitations. Posttreatment reassessment showed marked improvements, including lumbar spine AROM restored to full flexion at 70°, extension at 30°, lateral bending at 35° bilaterally, and rotation at 45° bilaterally. Functional tests normalized, with full squat depth and single-leg balance exceeding 30 seconds bilaterally. The stiffness and pain severity were rated from 5/10 to 0/10. At a 24-month follow-up, the patient reported high satisfaction with the conservative treatment approach, highlighting the reduction in symptoms and eventual return to competitive tennis as life-changing. He expressed gratitude for avoiding surgery and regaining his athletic career (Table 1).

**Table 1:** Symptom timeline AROM, active range of motion; LARF, lumbar apophyseal ring fracture; LE, lower extremity

Time point	Clinical events and symptoms	Pain score (0-10)	Functional measures
Month 0	Gradual onset of bilateral LE tightness and mild electric sensations during intensive tennis training	5/10	Lumbar AROM: flexion 30°, extension 15°, lateral bending 20°, rotation 25°; squat depth: 50%
Month 1	Symptoms persist despite rest; intermittent leg discomfort increases	5/10	Lumbar AROM unchanged; squat depth: 50%
Month 2	Presentation to clinic; neurological findings on exam; imaging ordered	5/10	Lumbar AROM unchanged; squat depth: 50%
Month 3	Imaging confirms LARF with disc herniation; conservative management started	4/10	Lumbar AROM unchanged; squat depth: 50%
Months 4-6	Gradual resolution of symptoms with chiropractic treatment and activity modification	2/10 (Month 4), 0/10 (Month 6)	Month 6: lumbar AROM: flexion 70°, extension 30°, lateral bending 35°, rotation 45°; squat depth: 100%
Month 12	Full return to competitive tennis, symptom-free	0/10	Lumbar AROM: full; squat depth: 100%; single-leg balance: >30 seconds bilaterally
Month 24	Remains asymptomatic at follow-up	0/10	Lumbar AROM: full; squat depth: 100%; single-leg balance: >30 seconds bilaterally

## Discussion

This case represents a rare documentation of successful conservative management of LARF with LDH in an adolescent athlete. While chiropractors have provided conservative care for disc herniations in the literature [8], this case is notable for demonstrating complete symptom resolution and a full return to high-level competitive tennis, outcomes that compare favorably to both similar conservative cases [9,10] and surgical series [6,9], where return to activity may take three to 12 months postoperatively. Our patient’s rapid reduction in pain and restoration of function, with symptom resolution and sports participation within six months, exceeded minimal clinically important difference thresholds reported in similar populations (a decrease of 2 points on a 10-point pain scale and a 10-15% improvement in function). Importantly, conservative management avoided surgical risks such as infection and neurologic injury, which are reported in 5-15% of operative cases.

Surgical intervention, such as posterior discectomy, fragment removal, or spinal fusion, remains standard for LARF with persistent neurological symptoms or failed conservative treatment, generally providing good symptom relief but with inherent risks, including dural tears, cerebrospinal fluid leakage, infection, and longer recovery periods that may limit adolescent athletic participation [2,4,6]. In contrast, our case reinforces that individualized conservative care, including chiropractic therapy and mechanical lumbar distraction, can achieve complete symptom resolution, restore full athletic function, and avoid perioperative complications. Notably, the patient remained symptom-free and active at 24 months posttreatment. This expands the scope of viable treatment options and suggests conservative care as a safe and effective alternative for selected patients [11,12], particularly when surgical risks outweigh benefits or noninvasive strategies are preferred.

The limitations of this single case report include its lack of generalizability and the challenge of comparing outcomes across studies due to heterogeneous clinical assessments and nonstandardized quantitative metrics, which reduces the strength of comparative claims. Larger studies with uniform outcome measures are needed to validate these findings. Diagnostic challenges, such as detecting apophyseal fractures on standard imaging, also remain a barrier to timely diagnosis and treatment. In this case, follow-up imaging was not performed due to the patient’s full recovery, but would have been considered if symptoms persisted or worsened; such imaging should be reserved for cases where clinical progress is uncertain or complications are suspected.

## Conclusions

This case highlights the successful conservative management of a LARF with central disc herniation (LDH) in an adolescent athlete, demonstrating that nonsurgical treatment can lead to complete symptom resolution and a full return to high-level athletic performance. Our results underscore that, in accordance with current clinical guidelines recommending conservative management for neurologically stable pediatric patients with LARF and disc herniation, close clinical monitoring without routine follow-up imaging can be effective, supporting individualized, evidence-based decision-making in this population while also highlighting the potential cost-effectiveness of a symptom-guided approach by reducing unnecessary imaging and surgical interventions and optimizing resource utilization in clinical practice. As the third chiropractic case report on LARF in PubMed, this study emphasizes the importance of exploring noninvasive treatment options for this rare condition. It underscores the need for early diagnosis, individualized care, and further research into conservative management strategies to provide clinicians with evidence-based alternatives to surgery.
